# A prospective study of the relationship between patient character and blood pressure in dental implant surgery

**DOI:** 10.1186/s40729-016-0054-2

**Published:** 2016-11-02

**Authors:** Masahiro Wada, Syunta Miwa, Tomoaki Mameno, Tohru Suganami, Kazunori Ikebe, Yoshinobu Maeda

**Affiliations:** Department of Prosthodontics, Gerodontology and Oral Rehabilitation, Osaka University Graduate School of Dentistry, 1-8 Yamadaoka, Suita, Osaka 565-0871 Japan

**Keywords:** Implant surgery, Personal traits, Blood pressure

## Abstract

**Background:**

Patients often suffer from physical and mental stress in dental implant surgery. The aim of this prospective study is to investigate the relationship between patient character and blood pressure in dental implant surgery.

**Methods:**

Fifteen patients were recruited for the present study. All patients had never received implant treatment in the past. To evaluate the patients’ personality trait, NEO-Five Factor Inventory (NEO-FFI) was used. All patients answered 50 questions at the first visit and divided in five dimensions: neuroticism, extraversion, openness, agreeableness, and conscientiousness. The index of physical stress was evaluated by blood pressure and pulse rate.

**Results:**

Ten females and five males (mean 55.5 ± 10.6 years) were evaluated in this study. A significant positive correlation was found between elevation rate of diastolic blood pressure/mean blood pressure and neuroticism score (rs *=* 0.584, 0.526, *p* < 0.05). On the other hand, there was no significant correlation between systolic blood pressure elevation and neuroticism score.

**Conclusions:**

In this limited study, there was significant correlation between neuroticism character and diastolic blood pressure or mean blood pressure rising in patients who received implant surgery.

## Background

Implant prosthesis has already become one of the treatment options for missing teeth. In addition, under the appropriate maintenance therapy, its longevity is equal or higher compared to the other prosthetic treatments. On the other hand, patients who want to receive the implant treatment cannot avoid the insertion surgery. In addition, if there are hard or soft tissue defects at the planned site, even an additional surgery is necessary [1, 2]. Therefore, patients who want to receive implant prosthesis often suffer from mental and physical stress in the surgical phase.

In many cases, we can reduce physical stress under the appropriate anesthesia and monitor general conditions during or just before implant surgery [3]. Mental stress, such as uneasiness or fear, sometimes caused blood pressure elevation. However, this symptom gradually decreases just only by the rest at the place and does not become the problem for implant surgery in most cases. In fact, Schwarz-Arad et al. reported that there is a strong correlation between uneasiness for treatment and the experience of pain at the operation that patients received in the past [4]. They also mentioned that the degree of uneasiness became highest just before the surgery and gradually decreased at the end of the surgery. Morino et al. reported that one of the stress markers, chromogranin A level of salivary, was high before implant surgery [5].

However, in some patient cases, many dentists experienced the uncontrolled patients even if they provided enough informed consent and appropriately managed the general condition at the surgery; as a result, they had to stop or cancel the implant surgery. One of these reasons is thought to be the influence of the patient’s character. Pavek et al. concluded that mean levels of blood pressure of 85 men during a whole day are slightly affected by particular personality traits [6]. Pilqrim et al. also mentioned that acute anxiety or uneasiness can lead to a transient rise in blood pressure [7]. However, there are few reports about these relationships in dental treatment, especially in implant treatment. The aim of this study is to investigate the relationship between patient character and blood pressure in dental implant surgery.

## Methods

Fifteen patients were recruited for the present study. All the patients had never received implant treatment in the past. The patients were not accepted into this study if they met any of the following exclusion criteria: (1) experience of implant treatment in the past, (2) general contraindications to implant surgery, (3) moderate or severe hypertension or cardiovascular system disease, and (4) mental disease. All the patients were informed about the purpose of this study and provided their signed informed consent.

To evaluate the patients’ personality trait, NEO-Five Factor Inventory (NEO-FFI) was used. All the patients answered 50 questions at the first visit and scored 0–40 in five dimensions: neuroticism, extraversion, openness, agreeableness, and conscientiousness. The index of physical stress was evaluated by blood pressure and pulse rate. All the patients’ blood pressure (systolic blood pressure (SBP), diastolic blood pressure (DBP), mean blood pressure (MBP)) and pulse rate (PR) were measured by a biological information monitor (Moneo BP-A308, OMRON COLIN Co., Tokyo, Japan) at the first visit, entering into the operation room without local anesthesia given before and after the surgery. Elevation rates (ER) of each blood pressure and PR were calculated. Rate pressure product (RPP) was also calculated by SBP and PR.

All the patients received implant surgery under local anesthesia (2 % lignocaine with 1:80,000 epinephrine; DENTSPLY International Inc., PA, USA), and a one- or two-stage surgical approach was selected according to the initial torque values and resonance frequency analysis (RFA). Screw-type dental implants (GENESiO Plus, GC Company, Tokyo, Japan) were used in all the patients.

SBP_ER, DBP_ER, MBP_ER, PR_ER, and RPP_ER and five dimensions of NEO-FFI were used as correlated variables. For statistical analysis, Spearman’s rank correlation was used and *p* < 0.05 was considered statistically significant.

This study was approved by the Ethics Committee of Osaka University Graduate School of Dentistry (H24-E16-1).

## Results

Ten females and five males (mean 55.5 ± 10.6 years) were evaluated in this study (Table 1). Tables 2 and 3 show the patients’ blood pressure, pulse rate, and NEO-FFI scores. Average values of blood pressure and pulse rate at the first visit were 121.2 mmHg; SBP, 74.9 mmHg; DBP, 90.4 mmHg; MBP, 81.6 bpm; PR, RPP, 9791 bpm *×* mmHg and 143.0 mmHg; SBP, 87.1 mmHg; DBP, 105.7 mmHg; MBP, 69.0 bpm; PR, RPP, 9859 bpm × mmHg just before the surgery. A significant positive correlation was found between DBP_ER/MBP_ER and neuroticism score (rs *=* 0.584, 0.526, *p* < 0.05). There was no significant correlation, but the same tendency was found with extraversion score (rs *=* −0.478, −0.499, *p* = 0.072, 0.058) in DBP_ER and MBP_ER. On the other hand, there were no significant correlation between SBP_ER, PR_ER, RPP_ER, and neuroticism and extraversion scores. In addition, other personality traits have no correlation with blood pressure, pulse rate, and RPP (Table 4).

**Table 1:** Overview of the patients’ data. All indexes were elevated just before the implant surgery

No.	Sex	Age	SBP (mmHg)	DBP (mmHg)	MDP (mmHg)	PR (bpm)	RPP (bpm × mmHg)
First visit							
1	Female	63	91	52	65.0	68	6188
2	Male	66	155	86	109.0	96	14,880
3	Female	36	110	65	80.0	70	7700
4	Male	54	139	92	107.7	70	9730
5	Male	59	130	80	96.7	65	8450
6	Female	41	100	70	80.0	72	7200
7	Female	62	105	80	88.3	76	7980
8	Male	72	130	90	103.3	67	8710
9	Female	69	148	88	108.0	84	12,432
10	Female	51	120	70	86.7	65	7800
11	Male	68	110	70	83.3	73	8030
12	Female	46	115	65	81.7	65	7475
13	Female	53	109	76	87.0	72	7848
14	Female	66	135	65	88.3	74	9990
15	Female	59	117	80	92.3	66	7722
	Mean (SD)	57.6 ± 10.3	121.2 ± 17.5	74.9 ± 10.9	90.4 ± 12.1	72.2 ± 8.3	8809.0 ± 2224.0
Just before surgery							
1	Female	63	124	65	84.7	70	8680
2	Male	66	175	98	123.7	103	18,025
3	Female	36	128	81	96.7	108	13,824
4	Male	54	141	92	108.3	84	11,844
5	Male	59	143	99	113.7	63	9009
6	Female	41	133	90	104.3	78	10,374
7	Female	62	156	91	112.7	76	11,856
8	Male	72	116	74	88.0	61	7076
9	Female	69	198	117	144.0	86	17,028
10	Female	51	118	76	90.0	77	9086
11	Male	68	148	83	104.7	80	11,840
12	Female	46	131	75	93.7	66	8646
13	Female	53	132	82	98.7	75	9900
14	Female	66	159	96	117.0	76	12,084
15	Female	59	145	90	108.3	63	9135
	Mean (SD)	57.6 ± 10.3	143.0 ± 21.3	87.1 ± 12.4	105.7 ± 14.9	77.7 ± 13.6	11,227.1 ± 3108.0

**Table 2:** Elevation rate (ER) of each index. Blood pressure just before the surgery rose around 20 %

	Elevation rate				
No.	SBP (%)	DBP (%)	MDP (%)	PR (%)	RPP (%)
1	36.3	25.0	30.3	2.9	40
2	12.9	14.0	13.5	7.3	21
3	16.4	24.6	20.8	54.3	80
4	1.4	0.0	0.6	20.0	22
5	10.0	23.8	17.6	−3.1	7
6	33.0	28.6	30.4	8.3	44
7	48.6	13.8	27.5	0.0	49
8	−10.8	−17.8	−14.8	−9.0	−19
9	33.8	33.0	33.3	2.4	37
10	−1.7	8.6	3.8	18.5	16
11	34.5	18.6	25.6	9.6	47
12	13.9	15.4	14.7	1.5	16
13	21.1	7.9	13.4	4.2	26
14	17.8	47.7	32.5	2.7	21
15	23.9	12.5	17.3	−4.5	18
Mean (SD)	19.1 ± 15.6	17.4 ± 14.6	17.8 ± 13.0	7.6 ± 15.0	28.3 ± 22.5

**Table 3:** NEO-FFI score of the patients. This questionnaire can evaluate the patient’s personal trait in five categories

	NEO-FFI score				
No.	Neuroticism	Extraversion	Openness	Agreeableness	Conscientiousness
1	27	20	27	32	26
2	14	27	35	24	30
3	23	28	25	34	27
4	19	30	28	30	19
5	33	10	23	30	27
6	22	25	32	30	18
7	19	27	30	30	28
8	14	32	25	29	28
9	32	23	24	25	28
10	24	24	34	36	26
11	17	29	30	29	33
12	15	35	33	37	38
13	9	28	30	41	31
14	20	21	34	25	21
15	20	15	26	30	32
Mean (SD)	20.6 ± 6.4	25.6 ± 6.3	29.3 ± 3.8	30.9 ± 4.5	27.1 ± 5.1

**Table 4:** The correlation between personal traits and blood pressure. A significant positive correlation were found between DBP_ER/MBP_ER and neuroticism score. In addition, there was the same tendency between DBP_ER/MBP_ER and extraversion score

		Neuroticism	Extraversion	Openness	Agreeableness	Conscientiousness
SBP_ER (%)	rs	0.158	−0.272	−0.047	−0.055	0.149
	*p*	0.575	0.327	0.869	0.847	0.596
DBP_ER (%)	rs	0.584**	−0.478*	−0.023	−0.271	−0.275
	*p*	0.022	0.072	0.934	0.328	0.322
MBP_ER (%)	rs	0.526**	−0.499*	−0.077	−0.299	−0.201
	*p*	0.044	0.058	0.784	0.279	0.472
PR (%)	rs	0.050	0.250	0.334	0.200	−0.368
	*p*	0.859	0.368	0.224	0.474	0.177
RPP (bpm × mmHg)	rs	0.102	0.059	−0.011	0.029	−0.122
	*p*	0.717	0.834	0.970	0.918	0.665

*<0.05; **<0.1

## Discussion

The blood pressure is considered to be affected by many factors, including physical and psychological stress in dental treatment. From a physical aspect, there are many reports about the relationship between blood pressure and local anesthesia or pain [8–11].

In fact, Tsuchihashi et al. reported that there was a correlation between increased blood pressure and infiltrated anesthesia amount [12].

On the other hand, there are not so many studies about the blood pressure change affected by the psychological aspect.

In this study, we found that neuroticism is significantly correlated with DBP and MBP increase. Ploubidis et al. reported that a person with a high degree of neuroticism assessed with the Eysenck Personality Inventory tended to self-select situations likely to lead to adversity and distress [13]. Guasti et al. also found that there was a positive correlation between provoked stress and blood pressure change using mental arithmetic stress test [14]. In addition, Eli et al. found that a patient’s anxiety was highest immediately before a surgical procedure compared to immediately post-operatively and at 4 weeks post-operative follow-up [15]. These results are similar to our findings. It is thought that increase of blood pressure sometimes causes hematoma formation and increases circulation blood volume. As a result, the burden to physical condition including cardiac function is expected. Rate pressure product (RPP), which had no correlation with personal traits in this study, is also a reliable predictor of myocardial oxygen consumption, and RPP >12,000 bpm × mmHg is associated with myocardial ischemia [16–18].

There were no severe problems during the implant surgery because the patients in this study did not have moderate or severe hypertension or cardiovascular system disease. However, we should pay enough consideration to elder people or patients who have these systemic diseases and neuroticism or extraversion character during implant surgery. Applications such as nitrous oxide or intravenous sedation may be desirable for these patients. In fact, Ichinobe et al. reported that intravenous sedation stabilized measureable changes in blood pressure and pulse rate due to fear and anxiety about dental treatment [19].

On the other hand, there was no correlation between SBP and neuroticism character. For this reason, it is thought that the range of SBP is usually wide compared to DBP. However, there are some reports about the correlation between SBP and patients’ personality trait [20]. Besides, the excessive rise in SBP during the operation may adversely affect the condition of the patients. Therefore, a further study about this relationship is necessary.

Measurement of a stress marker such as cortisol or amylase in saliva is one of the useful methods of stress evaluation. Umeanula reported that patients who received tooth extraction had increased cortisol concentration in saliva and concluded that evaluation of a stress marker is a simple, effective method for stress evaluation [21]. However, measuring the stress marker in saliva costs time and is expense. Alternatively, we used NEO-FFI for the evaluation of patients’ personality trait. NEO-FFI is a simple and easy questionnaire consisting of 50 questions, and we can easily analyze the character tendency of the patients into five dimensions (neuroticism, extraversion, openness, agreeableness, and conscientiousness). Yoshimura et al. revealed the validity of NEO-FFI using a large community sample in Japan [22]. Schwartz-Arad et al. reported that there was a positive correlation between the degree of anxiety and patient’s expectation to experience pain during the implant surgery and concluded that in a stressful pre-surgical situation, the ability to process relevant information may be severely impaired and should not be given to patients just before the surgery [4]. Therefore, this non-invasive and simple method for evaluation of a patient’s character is very useful. Besides, it is thought that the results of NEO-FFI, especially neuroticism and extraversion tendency, serve as a stress reference in not only implant treatment but also general dental treatment.

## Conclusions

In this limited study, there was a significant correlation between neuroticism character and diastolic blood pressure or mean blood pressure rising in patients who received implant surgery.
